# Negative symptoms correlate with altered brain structural asymmetry in amygdala and superior temporal region in schizophrenia patients

**DOI:** 10.3389/fpsyt.2022.1000560

**Published:** 2022-09-26

**Authors:** Zetao Huang, Dun Ruan, Bingjie Huang, Tianhang Zhou, Chuan Shi, Xin Yu, Raymond C. K. Chan, Yi Wang, Chengcheng Pu

**Affiliations:** ^1^Peking University Sixth Hospital, Peking University Institute of Mental Health, NHC Key Laboratory of Mental Health (Peking University), National Clinical Research Center for Mental Disorders (Peking University Sixth Hospital), Beijing, China; ^2^Neuropsychology and Applied Cognitive Neuroscience Laboratory, CAS Key Laboratory of Mental Health, Institute of Psychology, Chinese Academy of Sciences, Beijing, China; ^3^Department of Psychology, University of Chinese Academy of Sciences, Beijing, China

**Keywords:** schizophrenia, predominantly negative symptoms, cortical thickness, asymmetry, amygdala

## Abstract

Negative symptoms play an important role in development and treatment of schizophrenia. However, brain changes relevant to negative symptoms are still unclear. This study examined brain structural abnormalities and their asymmetry in schizophrenia patients and the association with negative symptoms. Fifty-nine schizophrenia patients and 66 healthy controls undertook structural brain scans. Schizophrenia patients were further divided into predominant negative symptoms (PNS, *n* = 18) and non-PNS (*n* = 34) subgroups. Negative symptoms were assessed by the Negative Symptom Assessment (NSA). T1-weighted images were preprocessed with FreeSurfer to estimate subcortical volumes, cortical thickness and surface areas, asymmetry Index (AI) was then calculated. MANOVA was performed for group differences while partial correlations in patients were analyzed between altered brain structures and negative symptoms. Compared to healthy controls, schizophrenia patients exhibited thinner cortices in frontal and temporal regions, and decreased leftward asymmetry of superior temporal gyrus (STG) in cortical thickness. Patients with PNS exhibited increased rightward asymmetry of amygdala volumes than non-PNS subgroup. In patients, AI of cortical thickness in the STG was negatively correlated with NSA-Emotion scores (*r* = −0.30, *p* = 0.035), while AI of amygdala volume was negatively correlated with NSA-Communication (*r* = −0.30, *p* = 0.039) and NSA-Total scores (*r* = −0.30, *p* = 0.038). Our findings suggested schizophrenia patients exhibited cortical thinning and altered lateralization of brain structures. Emotion and communication dimensions of negative symptoms also correlated with the structural asymmetry of amygdala and superior temporal regions in schizophrenia patients.

## Introduction

Clinically, negative symptoms contribute to poor social functioning outcomes in schizophrenia patients and unneglectable burden on families, society and health-care system (1). The Positive and Negative Syndrome Scale (PANSS) (2), Scale for the Assessment of Negative Symptoms (SANS) (3), as well as the Negative Symptom Assessment (NSA) (4) are commonly used for assessing negative symptoms worldwide. The NSA was developed to overcome some of the limitations assessing negative symptoms by the PANSS and SANS, such as including items that measure cognitive functioning (e.g., abstract thinking for PANSS, attention deficit for SANS), which are now recognized to be conceptually distinct from negative symptoms (5). In 2005, the National Institute of Mental Health-Measurement and Treatment Research to Improve Cognition in Schizophrenia (NIMH-MATRICS) consensus has agreed on five domains of negative symptoms including alogia, asociality, anhedonia, avolition and blunted affect (6). Since then, new assessments including the Brief Negative Symptom Scale (BNSS) (7) and the Clinical Assessment Interview for Negative Symptoms (CAINS) (8) have been developed and validated in clinical studies. Although researchers are getting aware of the importance of assessing negative symptoms in clinical treatment, there is still lack of studies specifically investigating brain mechanism underlying negative symptoms in schizophrenia.

Nowadays, several neuroimaging studies have been conducted to examine the brain structural changes in schizophrenia. Widespread thinner cortices have been consistently observed with regional specificity in frontal and temporal regions (9, 10). In addition, reduced gray matter volume (GMV) in subcortical regions has been observed in schizophrenia (11), with large-scale meta-analyses detecting a smaller thalamus, nucleus accumbens, and intracranial volume (ICV) as well as larger volumes of pallidum and lateral ventricle (12). Some studies suggested significant correlations between brain structural changes and severity of negative symptoms in schizophrenia patients (13, 14). Considering the heterogeneity of clinical manifestations, researchers have also investigated the differences between subgroups of schizophrenia patients with and without prominent, predominant or persistent negative symptoms (15). In line with this approach, reduction of gray matter volume and cortical thickness in frontal and temporal lobes have been linked to negative symptoms (16–18). An ALE meta-analysis also suggested that reduced gray matter volume was found in caudate and frontal lobe in schizophrenia with persistent negative symptoms (19). Together, these findings suggested that negative symptoms may relate to specific brain structural changes in schizophrenia and further investigations are necessary.

Laterality or asymmetry is an important characteristic of human brain. Anatomically, protrusions of the right frontal and the left occipital regions lay over the midline on an overall hemisphere level (known as “Yakovlevian torque”) in healthy people (20, 21). The degree of lateralization could help us better understand structural asymmetry and hemispheric dominance of specific brain regions. A recent meta-analysis further reported that left hemisphere exhibited a thicker cortex but smaller surface area relative to the right hemisphere in general population (22). Schizophrenia has been considered as a lateralized brain disease and studies have reported an increased leftward laterality of striatal volume, including pallidum, and reduced leftward laterality of thalamic volume (11). In addition, previous studies also suggested that abnormal asymmetry of brain volumes are related to negative symptoms (23–25). We acknowledged some studies examined the brain volumes/cortical thickness in patients with prominent, predominantly, or persistent negative symptoms and highlighted abnormalities specific to one hemisphere such as left amygdala, left superior temporal gyrus and right hippocampus, etc. (17, 26), however, the associations between abnormal asymmetry in brain structural measures and different dimensions of negative symptoms in schizophrenia is still awaiting further investigation.

In this study, we aimed to investigate the brain structural changes in schizophrenia patients, including subcortical volumes, cortical thickness, surface area as well as asymmetry of brain structural measures, by comparing patients to healthy controls. We split patients into predominant negative symptoms (PNS) and non-PNS subgroups to explore the difference on brain structural changes and asymmetry. We also explored the correlations between altered brain structural measures and negative symptoms in the whole group of patients. Based on prior findings, we hypothesized that (1) compared with healthy controls, schizophrenia patients would exhibit reduced cortical thickness in frontal and temporal regions while the PNS subgroup would exhibit thinner cortex in frontal region compared to the non-PNS subgroup; (2) for brain asymmetry, schizophrenia patients, in particular the PNS subgroup, would exhibit altered lateralization relative to their counterparts. (3) severity of negative symptoms would correlate with brain structural and asymmetric changes in the entire group of schizophrenia patients.

## Materials and methods

### Participants

We recruited 59 clinically stable patients with schizophrenia from Peking University Sixth Hospital, aged from 18 to 55. The diagnosis was ascertained by experienced psychiatrists using the International Classification of Diseases and Related Health Problems 10th Revision (ICD-10) (27). Healthy controls (n=66) were recruited from the neighboring communities. The MINI-International Neuropsychiatric Interview (M.I.N.I.) was used by qualified psychiatrists (28) to ascertain healthy controls did not suffer from personal or family history of psychiatric disorders. Exclusion criteria for all participants included (a) an estimated IQ below 70; (b) a history of head injury or neurological disorders; (c) brain structural abnormalities; (d) a history of substance use; (e) having contraindications for MRI scanning; and for patients only (f) a history of transcranial magnetic stimulation or electroconvulsive therapy in the past 12 weeks.

Patients were further split into subgroups with and without PNS based on severity of clinical symptoms measured by the Positive and Negative Syndrome Scale (PANSS) (2). Patients with PNS should meet two additional criteria: (a) PANSS negative subscale score ≥6 points over the PANSS positive subscale; (b) fewer than 3 items with score ≥3 (mild) on the PANSS positive subscale (19, 29). In total, 18 patients were classified to PNS subgroup and 34 to non-PNS subgroup.

The study was approved by the Ethic Committee of Peking University Sixth Hospital (Protocol 2014-30) and followed the Declaration of Helsinki. Written informed consent was obtained from all participants.

### Assessment for clinical symptoms

Negative symptoms were assessed using the Chinese Version of the NSA (4, 30, 31). The 15-item NSA comprises three factors, namely communication, emotion and motivation. It covers a wide range of negative symptoms and a single item was measured for the global severity of negative symptoms based on the interviewer's global impression of the patients. In addition, severity of clinical symptoms was assessed using the Chinese version of Positive and Negative Syndrome Scale (PANSS) (28).

### Acquisition and preprocessing of brain structural images

All participants undertook a structural brain scan in a 3T GE MR750 scanner at the Center for Neuroimaging, Peking University Sixth Hospital, Beijing, China. For each participant, *t*1-weighted images were acquired using three-dimensional fast spoiled-gradient recalled acquisition (3D-FSPGR) in 192 sagittal slices, with slice thickness = 1.0 mm, repetition time (TR) = 6.66 ms, echo time (TE) = 2.93 ms, inversion time = 450 ms, flip angle = 12°, matrix size = 256 × 256 mm, voxel size = 1 × 1 × 1 mm 3, field of view (FOV) = 256 mm. Participants were asked to lie down peacefully without head motion during scanning.

The FreeSurfer imaging analysis suite (v6.0) (32, 33) was used for cortical reconstruction and subcortical segmentation. Each participant's t1-weighted image was skull-stripped and registered to the Talairach space, then segmented into white matter/gray matter (WM/GM) tissues based on signal intensities of local and neighboring voxels. The cortical surface of each hemisphere was inflated to an average spherical surface to locate both the pial surface and the WM/GM boundary. Cortical thickness was calculated based on the gray/white boundary to the gray/CSF boundary at each vertex on the tessellated surface (32). Surface area was also estimated by surface deformation procedures and surface inflation according to the surface inflation (33). In terms of the image quality, original t1-weighted images were first checked to exclude individuals with large head motion. Then, subcortical segmentation, gray matter-white matter boundary, pial surfaces and skull-striped brain masks were visually inspected, and if necessary, brain mask image was manually edited to remove non-brain tissue before re-running the surface reconstruction. Finally, cortical parcellation was carried out (34) based on the Desikan–Killiany Atlas (35), regional cortical thickness and surface area for 68 regions in both hemispheres were extracted, respectively. In addition, volumes of subcortical structures, including the putamen, the caudate, the hippocampus, the amygdala, the thalamus, the nucleus accumbens and the pallidum, for each hemisphere as well as the intracranial volume (ICV) were extracted for each participant. In the current study, structural lateralization was defined as the ratio of the difference between the left and right volumes to the total volume of a brain region, measured by the Asymmetry Index (AI) for subcortical structures, reginal cortical thickness and surface area, respectively. Similar to previous study, the AI was calculated by the ratio of [(left–right)/(left + right)].calculated as [(left–right)/(left + right)], consisted with the previous study (11).

### Data analysis

Data analyses were performed using SPSS v21. First, we performed group comparison on subcortical volumes, regional cortical thickness and surface area, as well as the AIs of brain structural measures between patients and controls using multivariate analysis of variance (MANOVA), taking gender, age, years of education and ICV as covariates of no interests. In addition, MANOVAs were also conducted between PNS and non-PNS subgroups of schizophrenia patients to explore the differences on subcortical volumes, regional cortical thickness and surface area, as well as the AIs of brain structural measures taking gender, age, years of education and ICV as covariates. Bonferroni correction (which is *p* < 0.05/N, N denotes the number of dependent variables) was applied. In specific, threshold was set to *p* < 0.0035714 (0.05/14 regions) for subcortical volumes, *p* < 0.000735 (0.05/68 regions) for cortical thickness or surface area, *p* < 0.007143 (0.05/7 regions) for the AI of subcortical volumes and *p* < 0.0014706 (0.05/34 regions) for the AI of cortical thickness or surface area. Then, we performed partial correlation analyses between altered brain structural measures and severity of negative symptoms assessed by the NSA, taking gender, age, years of education and the ICV as covariates, significant threshold was set as *p* < 0.05.

## Results

### Demographic and clinical information

Table 1 shows the demographic and clinical information of the healthy controls and patients with schizophrenia. Two groups did not differ in gender proportions (*p* > 0.1) but schizophrenia patients were older (*p* < 0.05) and less educated (*p* < 0.05) than healthy controls. As shown in Table 1, the PNS and non-PNS subgroups did not differ in age, gender and length of education.

**Table 1:** Demographic information and clinical symptoms.

	**HC** ** (*N* = 66)**	**SZ** ** (*N* = 59)**	**PNS** ** (*N* = 18)**	**Non-PNS** ** (*N* = 34)**
Gender (% female)	0.59	0.63	0.56	0.68
Age (year)	23.14 ± 6.96	27.42 ± 8.60	27.28 ± 9.17	26.97 ± 8.28
Length of education (year)	15.23 ± 2.24	13.66 ± 3.41	12.94 ± 3.23	13.76 ± 3.38
Duration of illness ^a^ (year)	–	6.02 ± 5.54	5.40 ± 4.49	5.62 ± 4.85
Medication (chlorpromazine mg/day) ^b^	–	209.30 ± 217.41	260.94 ± 249.03	207.37 ± 195.55
NSA-15 total ^c^	–	38.66 ± 13.66	44.94 ± 12.74	33.53 ± 11.48
NSA-motivation	–	18.69 ± 6.43	22.17 ± 5.32	16.26 ± 5.88
NSA-communication	–	13.28 ± 6.10	15.17 ± 6.89	11.29 ± 4.39
NSA-emotion	–	6.69 ± 2.79	7.61 ± 2.64	5.97 ± 2.76
SANS-total ^c^	–	24.36 ± 25.69	34.11 ± 30.31	19.53 ± 21.65
SANS affective flattening	–	2.00 ± 1.59	2.72 ± 1.13	1.32 ± 1.51
SANS alogia	–	1.53 ± 1.44	1.94 ± 1.21	1.09 ± 1.42
SANS avolition–apathy	–	2.29 ± 1.56	3.06 ± 1.21	1.76 ± 1.58
SANS anhedonia–asociality	–	2.67 ± 1.43	3.67 ± 0.59	2.06 ± 1.41
SANS attention	–	0.52 ± 1.00	0.67 ± 1.08	0.41 ± 0.92
PANSS-total ^c^	–	59.72 ± 18.94	60.28 ± 14.94	57.59 ± 18.96
PANSS-positive	–	13.71 ± 5.50	10.22 ± 3.02	15.24 ± 5.61
PANSS-negative	–	17.60 ± 8.06	21.94 ± 7.24	14.21 ± 6.61
PANSS-general	–	28.66 ± 8.77	28.11 ± 7.33	28.15 ± 9.11

a Eighteen patients had missing data on this measure;

b Ten patients had missing data on this measure;

c Seven patients had missing data on these measures.

### Group comparison between schizophrenia and healthy controls

**Figure 1:**
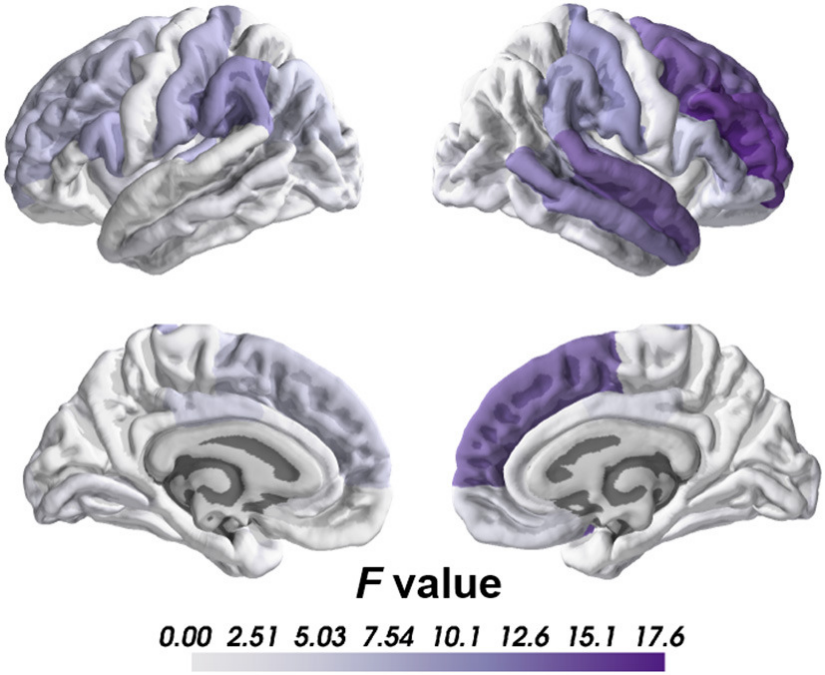
Differences between SCZ and HC groups on cortical thickness.

Compared to healthy controls, we found significant cortical thinning in schizophrenia group, including right caudal middle frontal, right middle temporal, right rostral middle frontal, right superior frontal and right superior temporal regions after Bonferroni corrections (Table 2; Figure 1). No significant group differences were found on subcortical volume or surface area after correction.

**Table 2:** Significant group differences between SCZ and HC on cortical thickness.

**Brain regions** ** (Right Hemisphere)**	**HC (*N* = 66)** ** M ±SE (mm)**	**SZ (*N* = 59** ** M ±SE (mm)**	***F***	***P***
Caudal middle frontal	2.727 ± 0.018	2.62 ± 0.019	14.99	0.00018
Middle temporal	2.965 ± 0.016	2.881 ± 0.017	12.46	0.00059
Rostral middle frontal	2.534 ± 0.016	2.435 ± 0.017	17.56	0.00005
Superior frontal	2.945 ± 0.016	2.857 ± 0.017	13.62	0.00034
Superior temporal	2.949 ± 0.016	2.854 ± 0.017	14.41	0.00023

Regards to the brain lateralization, patients with schizophrenia exhibited significantly increased AI of the cortical thickness in superior temporal gyrus than healthy controls (HC = −0.014, SCZ = 0.000, mean difference = 0.014, SE = 0.003, *F* = 19.28, *p* = 0.00002) indicating that cortical thickness of the superior temporal gyrus in healthy controls was rightward lateralized while patients did not show similar pattern.

### Comparison between PNS and non-PNS subgroups of schizophrenia

We did not find significant differences on brain structural measures between the PNS and non-PNS subgroups after Bonferroni correction. Concerning lateralization of brain structural measures, the PNS subgroup exhibited significantly reduced AI of amygdala volume than the non-PNS subgroup (PNS = −0.077, non-PNS = −0.035, mean difference = 0.031, SE = 0.010, *F* = 9.038, *p* = 0.00427), suggesting that PNS subgroup showed more rightward lateralization than non-PNS subgroup.

### Correlations with severity of negative symptoms

**Figure 2:**
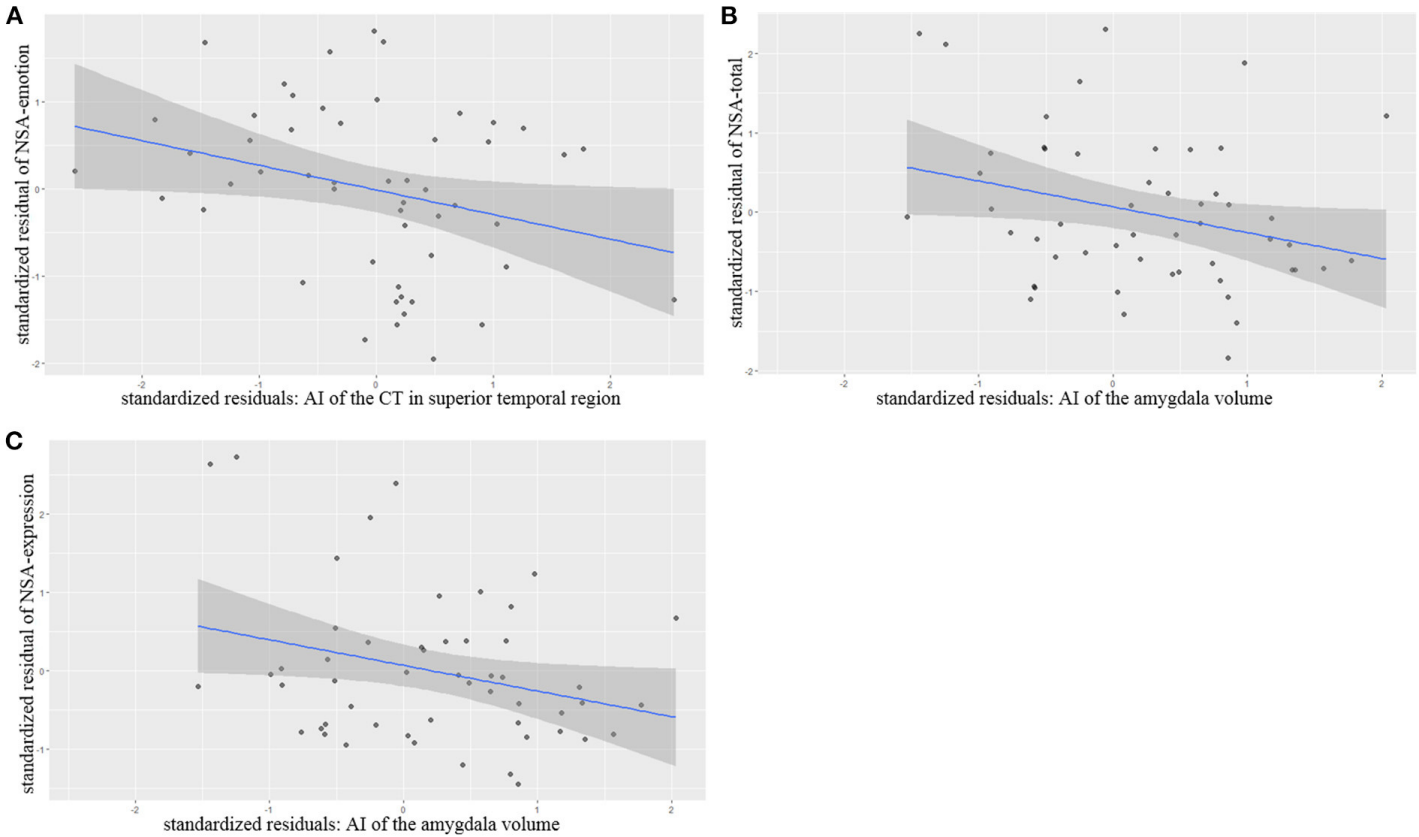
**(A–C)** Partial correlation between brain structural changes and NSA scores.

Partial correlation analysis controlling for age, gender, length of education and the ICV as covariates of no interests showed that AI of the superior temporal thickness correlated significantly and negatively with NSA-emotion scores (*r* = −0.302 *p* = 0.035), AI of the amygdala volumes also correlated negatively with NSA-total scores (*r* = −0.300 *p* = 0.039) and NSA-Communication (*r* = −0.301 *p* = 0.038) (Figure 2).

## Discussion

In this study, we examined brain structural changes in patients with schizophrenia and its correlations with negative symptoms. We found that schizophrenia patients exhibited widespread cortical thinning, especially in the frontal and temporal regions of right hemisphere, and altered lateralization of cortical thickness in the superior temporal gyrus. Patients with PNS and non-PNS did not differ in subcortical volumes, cortical thickness and surface area. However, patients with PNS exhibited increased rightward lateralization of amygdala volume compared to patients with non-PNS. Severity of negative symptoms also correlated with the observed brain structural changes in schizophrenia patients, including lateralization of cortical thickness in superior temporal gyrus and amygdala volume.

The findings of the widespread cortical thinning in frontal and temporal regions in schizophrenia patients are consistent with previous studies (9, 10, 36). Van Erp et al.'s meta-analysis (2018) showed that the largest effect sizes of cortical thinning appeared in frontal and temporal lobes. Both frontal and temporal lobes are crucial for high-level cognitive function, frontal lobe plays important role in executive control, emotional and social processes (37, 38), while temporal lobe is involved in various cognitive functions including auditory, semantic, language and social information processing (39, 40). In patients with schizophrenia, previous studies suggested that structural changes in frontal and temporal lobes might be related with their impairments in executive function, hallucination and negative symptoms (36, 41, 42). Although previous studies indicated that thinner cortex may correlated with more severe of negative symptoms (10), we did not find significant correlation in our sample. In addition, van Erp et al. (10) also found smaller surface area in schizophrenia with a relatively small effect size (Cohen's d around −0.25). Considering our sample size of schizophrenia is relatively small and Bonferroni multiple comparison correction was applied, it makes sense that we only found thinner cortex in frontal and temporal lobe regions, but no significant differences were found in group comparison on surface area.

Structural lateralization changes in particular regions are found in our research. Hemispheric lateralization is a characteristic organization principle observed in vertebrate brains (43), highly associated with the development of cognitive functions, language and so on from an evolutionary point of view. The development of hemispheric lateralization derives from a series of genetic, environmental (such as light) and physiological (such as hormone) factors (44). Crow et al. has proposed that schizophrenia is a mental disorder that stems from the failure of normal hemispheric lateralization in the temporal lobe region (45). By far, different dimensions of manifestations in schizophrenia have been linked with abnormal lateralization. For example, schizophrenia patients showed a different lateralization pattern from the healthy controls (11, 23–25), and these differences are associated with positive, negative symptoms. Consequently, hemispheric lateralization might serve as a potential biomarker for schizophrenia, which provide a chance for clinician to recognize and intervene the disease. Meta-analytic integration and large-scale multi-center studies are needed before any final conclusions can be drawn. Regarding the altered asymmetry of cortical thickness in the superior temporal gyrus (STG) in schizophrenia patients as a whole group compared to healthy controls, we observed healthy controls exhibited a rightward lateralization of cortical thickness in the superior temporal gyrus, but schizophrenia patients did not exhibit obvious asymmetry in this region. This difference on the asymmetry of the STG may related to the specificity of cortical thinning in right hemisphere. The STG is a long region located along the Sylvian fissure dorsally and superior temporal sulcus ventrally, participating in the auditory processing, language production and self-monitoring. Studies consistently suggested altered laterality of the STG (include its subregions) in patients with schizophrenia (46, 47), and asymmetry of the STG has been addressed to be a potential biomarker for schizophrenia recently (48). Clinically, aberrant asymmetry in the STG has been repeatedly associated with positive symptoms such as auditory hallucinations and thought disturbances (49), which is consistent with the proposition depicting dysfunction of the primary auditory cortex in the anterior and middle STG. However, its relationship with negative symptoms remains unclear. In our study, partial correlation analysis indicated that altered asymmetry of the STG is associated with factor scores of the NSA-Emotion in schizophrenia. It implied that the altered asymmetry of the STG might play an important role in emotion dimension of negative symptoms.

Although previous studies showed a rightward asymmetry of amygdala volume in both healthy controls and schizophrenia patients, they did not find significant differences between schizophrenia patients and controls, or adolescents with and without subclinical psychotic experiences (11, 50). In our study, we did not find differences between the whole patient group and healthy controls on the asymmetry of amygdala volumes either. However, by comparing two subgroups of schizophrenia patients we found that the PNS subgroup exhibited increased rightward asymmetry of amygdala volume (AI = −0.077) than non-PNS subgroup (AI = −0.035). Amygdala is involved in the appraisal of external stimuli with regard to their emotional significance (51) and plays important roles in emotion recognition (52), especially prominent in negatively valued emotions like sadness, anger and fear (53). A series of studies observed reduced volumes of amygdala in schizophrenia patients (11, 54), including a largest-ever meta-analysis (12). Furthermore, it is proposed that the left amygdala is dominant for processing positive emotions while the right amygdala is prominent for processing negative emotions (55). There also seems to be associations between amygdala lateralization and emotional arousal (56) as well as the perceptual properties of stimuli (56, 57). These findings together indicate that the asymmetry of amygdala may serve as a potential biomarker of schizophrenia that specific to negative symptoms. The results of partial correlation in our study provide further evidence for this speculation, the AI of amygdala is negatively correlated with negative symptoms, including total scores and communication factor scores of the NSA. Evidence of the relationship between reduced amygdala volume and poor emotion recognition has also been found in schizophrenia, whilst asymmetric abnormality of amygdala was reported to be associated with negative symptoms in schizophrenia and ultra-high risk individuals (58). Emotion recognition is an important component of social cognition and closely related to negative symptoms and social function in schizophrenia patients (59, 60). However, few studies have investigated brain asymmetry in schizophrenia and its relationship with clinical symptoms. Future studies should pay more attention on the asymmetric changes of brain structure or functions in schizophrenia to clarify this issue.

This study has several limitations. First, the present sample size is relatively small, which might be a reason that we only observed altered cortical thickness in frontal and temporal lobe. Second, patients recruited in our study were at a chronic stage, the medication and duration of illness may confound the brain structural measures. Third, we did not include the second-generation assessment of negative symptoms such as CAINS and BNSS in the present study. Future study will benefit from these new generation tools to specifically assess psychopathology of negative symptoms in schizophrenia patients. The last but not the least, previous studies suggested that differences in age, gender and education level might confound the analyses of cortical thickness and structural lateralization between schizophrenia and healthy control groups (22, 61, 62). For example, compared to female participants, males show larger rightward asymmetry in surface area (22). Although we took these factors as covariates in the analysis to control the potential influences statistically, it would be better to have well-matched samples in future.

Taken together, this study investigated the brain structural changes and asymmetry in schizophrenia patients, PNS in particular, and showed the cortical thinning in frontal and temporal lobe. More importantly, our findings suggested that emotion and communication dimensions of negative symptoms may associate with the altered asymmetry of brain structure in superior temporal and amygdala regions.

## Data availability statement

The original contributions presented in the study are included in the article/supplementary material, further inquiries can be directed to the corresponding author/s.

## Ethics statement

The studies involving human participants were reviewed and approved by Peking University Sixth Hospital Ethics Committee. The patients/participants provided their written informed consent to participate in this study.

## Author contributions

CP and YW designed the study, obtained funding, and supervised the study. ZH and DR performed the data analysis and interpretation, as well as the report writing. XY and RC conducted the research design and the manuscript writing. BH, TZ, and CS participated in the collection of data. All authors contributed to the article and approved the submitted version.

## Funding

This study was supported by National Science Fund China (No. 82171500), Key Program of Beijing Science and Technology Commission (No. D171100007017002), the Capital's Funds for Health Improvement and Research (CFH, No. 2018-4-4116), National Key R&D Program of China (2018YFC1314200), and the National Key Project of Scientific and Technical Supporting Programs (2007BAI17B04). YW was supported by the National Natural Science Foundation of China (31871114) and the CAS Key Laboratory of Mental Health, Institute of Psychology. The funders had no role in study design, data collection and analysis, decision to publish or preparation of the manuscript. We thank all the participants in this study.

## Conflict of interest

The authors declare that the research was conducted in the absence of any commercial or financial relationships that could be construed as a potential conflict of interest.

## Publisher's note

All claims expressed in this article are solely those of the authors and do not necessarily represent those of their affiliated organizations, or those of the publisher, the editors and the reviewers. Any product that may be evaluated in this article, or claim that may be made by its manufacturer, is not guaranteed or endorsed by the publisher.
